# Preferences for work arrangements: A discrete choice experiment

**DOI:** 10.1371/journal.pone.0254483

**Published:** 2021-07-12

**Authors:** Peter Valet, Carsten Sauer, Jochem Tolsma

**Affiliations:** 1 Department of Sociology, University of Bamberg, Bamberg, Germany; 2 Department of Sociology, Bielefeld University, Bielefeld, Germany; 3 Department of Sociology, University of Groningen, Groningen, The Netherlands; 4 Department of Sociology, Radboud University Nijmegen, Nijmegen, The Netherlands; University of Western Australia, AUSTRALIA

## Abstract

This study investigates individual preferences for work arrangements in a discrete choice experiment. Based on sociological and economic literature, we identified six essential job attributes—earnings, job security, training opportunities, scheduling flexibility, prestige of the company, and gender composition of the work team—and mapped these into hypothetical job offers. Out of three job offers, with different specifications in the respective job attributes, respondents had to choose the offer they considered as most attractive. In 2017, we implemented our choice experiment in two large-scale surveys conducted in two countries: Germany (N = 2,659) and the Netherlands (N = 2,678). Our analyses revealed that respondents considered all six job attributes in their decision process but had different priorities for each. Moreover, we found gendered preferences. Women preferred scheduling flexibility and a company with a good reputation, whereas men preferred jobs with high earnings and a permanent contract. Despite different national labor market regulations, different target populations, and different sampling strategies for the two surveys, job preferences for German and Dutch respondents were largely parallel.

## Introduction

In recent decades, increasing international competition, emerging markets, and shorter economic cycles, coupled with demographic changes, high unemployment rates, and restrictive employment regulations, have put many Western economies under pressure [1]. Some have responded by implementing deregulation policies. Intended to improve international competitiveness, deregulation allows employers to use their workforce more flexibly under changing market conditions [2, 3]. Consequently, the overall share of non-standard work arrangements—such as part time work, temporary employment, on-call work—has increased considerably [4]. At the same time as employers are shifting to atypical contracts for economic reasons, employees are demanding new and often more flexible work arrangements that align with their individual circumstances [5, 6]. Reasons for shifting employee demands include the ongoing individualization of the life-course [7, 8], the emergence of new family or non-work arrangements [9], and a growing interest in personal development and life-long learning [10]. Recent literature suggests, people often do not consider high earnings to be the main reason for choosing a job [11]. Instead, they are increasingly looking for a job that provides flexible working hours [12], a job they consider meaningful [13], a job that gives them a sense of belonging [14], or one that provides possibilities for personal development [15].

Today’s employers try to offer work arrangements that fit employees’ demands. Thus, a profound understanding of those demands, including and beyond earnings, is essential for employers as they compete with others for qualified personnel. A broader knowledge of the relative importance of job attributes is also crucial for research on labor processes and organizational inequality. While there seems to be broad consensus that “good” jobs are not only a matter of high earnings and job security [16], there is little evidence on the preference order of various job attributes and the extent to which employees focus more on flexible working hours or further training opportunities and less on high earnings. It would also be useful to know if such preferences are shared by most people or are subgroup specific. For example, theories on gendered socialization and traditional gender roles suggest men and women differ considerably in their preferences for extrinsic and intrinsic job traits [17, 18].

Thus, we set out in this study to investigate people’s preferences for specific job attributes using a discrete choice design. To learn which job attributes drive people’s job decisions, we constructed sets of three hypothetical job offers in which we experimentally varied the job attributes. Out of these three offers, respondents chose the one they considered most attractive. Based on various strands of the literature, we identified six job attributes that people are likely to consider when opting for or against a job: level of earnings, job security, options for further training, scheduling flexibility, the reputation of the company, and the gender composition of the work team. We implemented our choice experiment in two large-scale surveys in 2017—one in Germany and the other in the Netherlands.

Our study contributes to the literature in several ways. Discrete choice designs are particularly suitable to investigate how people deliberate between different alternatives. Thus, our discrete choice experiment provided an empirical test of people’s preferences for different job attributes. We studied the importance of these attributes for all respondents in concert and separately for men and women to explore how women and men differ in their decision processes. Moreover, the implementation of our choice experiment in surveys in two countries—Germany and the Netherlands—allowed us to investigate the consistency of evaluation patterns, not only between social groups, but also in different macro-structural contexts. The largely parallel results speak for their robustness and provide solid evidence of people’s preference structure for different job attributes.

### Individual job preferences

To understand which job attributes people consider more salient when opting for or against a job alternative, it is necessary to identify the most pertinent job attributes in a more general context. For this, the sociology of work literature provides a good starting point [16, 19]. This literature offers comprehensive theoretical and empirical evidence of the potential importance of specific job characteristics and builds on insights from economic and sociological theory and research.

From this literature, we identified six aspects that people consider most relevant: (1) earnings, (2) job security, (3) opportunities for further training, (4) scheduling flexibility, (5) prestige of the company, and (6) the gender composition of the work team. In the following, we elaborate on these six key job attributes and discuss their expected impacts on job decisions. We complement this review with a discussion of why men and women might differ in how they value a particular job attribute. Our gendered expectations mostly draw on theories of gendered socialization and traditional gender roles [15, 17, 18].

First, the level of earnings is a main concern of job seekers [20]. Classical micro economic theory defines the level of earnings as the most important aspect of a job, and people consistently try to maximize their earnings [21]. High earnings not only contribute directly to employees’ material well-being; they also provide social status. Research on subjective well-being suggests individual life satisfaction increases with the level of earnings [22, 23]. More recent studies, however, show positive but declining marginal effects of earnings on subjective well-being [24–26]. While this might indicate that the level of pay is not as important as previously assumed, meta-analytic studies of motivational initiatives point to the level of pay as the most effective employee motivator [20, 27]. Some scholars discuss gendered preferences for high earnings and argue that due to early gendered socialization, women are more intrinsically oriented, and men are more extrinsically motivated. Accordingly, men value high pay more than women, who consider other aspects of a job more important [17, 18, 28]. This argument aligns with a care-rationale reasoning, as women more often work in the low-paying care-service sector [29]. Overall, the various strands of literature suggest jobseekers value high earnings but are somewhat ambivalent about whether high earnings are the most important job characteristic. Based on the literature, we expected the opportunity to earn more in one job than another would be a crucial factor in the decision-making process for a specific job offer. Moreover, we expected that high pay would be more important for men than for women.

Second, the sociology of work literature argues job security determines perceived job quality. While being more individualistic and flexible than employees in the past, people still value the power to decide on their own whether they want to keep or change a job. Many national and comparative studies show employees frequently rank job security as most important when asked to state their preferences for different aspects of work [15, 30–33]. Yet the implementation of deregulation policies has resulted in a deterioration of job security for many employees [34], with severe consequences. Studies show the fear of job loss is comparable to the psychological distress people experience when they actually lose a job [35, 36]. Apart from psychological consequences, insecurity affects behavior. Individuals with insecure or temporary jobs tend to postpone important life events such as family formation [37] or capital investment [38]. Job insecurity is especially prevalent among newly hired employees who may not have a permanent contract. For example, in Germany in 2018, 35 percent of all newly hired employees in the private sector and about 50 percent in the public sector had a temporary contract [39]. As permanent contracts provide more job security, we expected people’s sense of the value of a job offer would increase with contract duration, and they would therefore prefer longer-term offers. As the male breadwinner norm remains dominant in many Western countries—especially in conservative welfare states such as Germany [40]—we expected job security would be more important for men than for women.

Third, opportunities for training and improved qualifications are attractive to employees, as they facilitate their advancement in both internal and external labor markets. Some evidence indicates the detrimental effects of a temporary job are weaker if employers provide their employees with opportunities for further training [41]. Other studies report decreasing preferences for high earnings associated with higher levels of learning opportunities [42]. From a human capital perspective, there are two types of further qualifications: (a) general human capital that is transferable to positions outside the actual job, which, in turn, makes the employee more attractive for employers outside the current workplace; (b) specific human capital which is predominantly company specific and therefore mostly beneficial for the internal labor market. Investing in specific human capital increases promotion opportunities and chances for higher pay within the company but has limited value for other employers. Accordingly, we expected that people would generally value opportunities for further training. Moreover, we expected that both men and women would value general human capital over specific human capital, as it would increase their options in the larger labor market.

Fourth, scheduling flexibility allows people to better reconcile the work and non-work spheres of their lives. The decline of the male breadwinner model has led to new arrangements of aligning work schedules with household needs. Many studies suggest women are predominantly faced with these challenges [43–45], especially if there are young children in the household [46]. Employers are increasingly reacting to these flexibility demands and offering more family friendly work arrangements. While jobs traditionally had a fixed start and end (mostly nine to five), nowadays employers are increasingly offering more flexible schedules to provide better compatibility [47]. Some are also offering extra time off if needed. Based on the literature, we expected that extended scheduling flexibility would be important for many people when opting for or against a job offer. As women still do most of the reconciling of work and family needs, we expected scheduling flexibility would be more important for women than for men, especially with children in the household.

Fifth, beyond these individual job attributes, employees’ identification with a job is often driven by the reputation of the company they work for [48, 49]. The sociology of work literature highlights that pride and dignity are important for employees’ well-being and commitment [50]. Feelings of pride stem from their work and from the reputation of their company. This also resonates with work on organizational citizenship and extra-role behavior [51]. In contrast, employees may behave dysfunctionally and show more counterproductive work behavior in organizations with lower prestige [52]. Accordingly, we expected people’s preferences for a job would increase when the reputation of the company was higher. As the reputation of a company can be considered an intrinsic job preference or a signal of prestige, we had no expectation of whether it would be more important for women or for men.

Sixth, many employees work in gender segregated occupations [53] or on gender segregated teams [54]. Various studies in work and in social psychology have investigated the functionality and performance of teams and their gender composition, yielding mixed results [55, 56]. Studies focusing on the interactions at the workplace or the formation process in formal teams highlight the importance of the gender composition of work groups and teams [57, 58]. There is ample evidence of gender homophily, suggesting that women prefer working with other women and men prefer working with other men [59, 60]. One explanation is that people expect others who are like them to act more predictably, have similar interests, and be more trustworthy [61]. Other studies have not found a homophily bias, and some have even found the contrary [62]. We expected information on the gender composition of the work team would be important for people’s job decisions. Following the mainstream research, we expected a gender homophily bias in preferences, whereby people would choose a job offer when most co-workers were the same gender as they were.

### The present study

Our literature review revealed a number of job characteristics beyond earnings that attract people. We also learned that women and men may value these characteristics (e.g., scheduling flexibility) differently. In our choice experiment, we presented three job descriptions with varying job attributes side by side to respondents. In 2017, we implemented our choice experiment in two large-scale surveys, one conducted in Germany and the other in the Netherlands. Germany and the Netherlands are similar in many respects. Both are welfare states offering various social security (health, unemployment) and family (allowance for children, maternity leave) benefits. At the same time, there are remarkable differences. For example, paternity leave (or father-specific parental leave) benefits are far more generous in Germany (9 weeks) than in in the Netherlands (2 days) [63]. General labor market statistics show quite low unemployment rates in both countries in 2017 (GER: 3.8; NL: 4.9) [64] and a similar inequality in disposable income—measured by a Gini coefficient of 0.294 in Germany and 0.285 in the Netherlands [65]. However, there are remarkable differences in the countries’ temporary and part-time employment patterns. In 2017, 12.8 percent of all employment in Germany was temporary; in the Netherlands, this share was considerably higher, at 21.8 percent. Despite this dissimilarity, both countries show a huge age gradient in temporary employment. Among the employees younger than 25, more than 50 percent (GER: 52.6; NL: 58.8) had temporary contracts; for those between 55 and 64, it was below 10 percent (GER: 3.4; NL: 7.5) [66]. Patterns in part-time employment are even more dissimilar than those for temporary employment. In Germany, the part-time employment rate in 2017 was 22 percent, in the Netherlands 37 percent. In both countries, more women than men worked part-time. In Germany, 36 percent of all working women were part-timers, compared to 9 percent of all working men; in the Netherlands 56 percent of all working women were part-timers, compared to 19 percent of all working men [67].

The implementation of our choice experiment in countries with both basic similarities and considerable differences allowed us to investigate people’s preferences for certain job attributes and differences between social groups and also to verify our results in a cross-country replication.

## Materials and methods

### Respondents

The data for our study were collected in 2017 as part of two independent national surveys. The German data stem from the second wave of the employee panel survey “Legitimation of inequalities over the life-span” (LINOS-2). The target population of the first wave (LINOS-1) conducted in 2013 was employees subject to social security contributions. This included most private and public sector employees but excluded the self-employed and civil servants. Respondents for LINOS-1 were randomly sampled all over Germany. About 76 percent of the LINOS-1 respondents who consented to stay in the panel also participated in LINOS-2—representing 2,741 respondents. The LINOS survey was conducted as a multi-mode survey with random allocation to one of two modes: self-interviewing (by mail or online, depending on respondent’s preference) or personal computer-assisted interviewing. A detailed description of the data, the sampling procedure, and the materials used in the survey can be found in the technical reports [68–70]. As the discrete choice experiment was implemented only in LINOS-2, we restricted our analyses to this wave. For re-analysis purposes, the full dataset is available under the restriction of the German law for potentially sensitive data. Interested users must apply for data access, and the data can only be accessed on-site at the German Institute for Economic Research (DIW Berlin).

The Dutch data come from the Family Survey Dutch Population (FSDP) [71]. The FSDP is a large-scale survey that began in 1992 and has since been conducted at five-year intervals by the Sociology Department of Radboud University Nijmegen. All citizens of the Netherlands irrespective of their employment form the target population of the FSDP. Our discrete choice experiment was implemented in the 2017 wave. The 2017 FSDP wave consisted of 3,099 respondents who were members of the Longitudinal Internet Studies for the Social Sciences (LISS) Panel. The total LISS panel consisted of 4,500 households, comprising 7,000 individuals and was based on a probability sample of households drawn from the population register by Statistics Netherlands. The 2017 FSDP wave data is accessible through the LISS Data Archive [71].

Table 1 shows the arithmetic means, standard deviations (SD), minimums (Min.), and maximums (Max.) for key variables describing the two samples. Overall, we see that the samples differed only slightly. Looking at demographics, we see a slightly larger share of women, somewhat older respondents, a higher share of college degrees, and a higher share of respondents in the Dutch data. Obviously, the key difference between the two samples is the share of currently employed which, due to the discussed differences in the sampling, was much higher in Germany than in the Netherlands. In line with the OECD data on part-time employment [67], weekly working hours were higher in Germany than in the Netherlands.

**Table 1:** Description of the German and Dutch samples.

	Respondents from Germany				Respondents from the Netherlands			
	Mean	SD	Min.	Max.	Mean	SD	Min.	Max.
Gender (1 = woman)	0.52	-	0	1	0.54	-	0	1
Age	41.3	11.7	18	67	48.6	15.1	18	71
Kids in Household (1 = yes)	0.41	-	0	1	0.47	-	0	1
College/university degree (1 = yes)	0.33	-	0	1	0.41	-	0	1
Currently employed (1 = yes)	0.89	-	0	1	0.64	-	0	1
Working hours (weekly; if employed)	37.9	11.1	1	80	32.5	11.8	1	80
Gross earnings (monthly; if employed)	3406.6	3216.5	80	73000	2963.3	1801.8	75	16000
Job satisfaction (if employed)	6.90	2.20	0	10	7.27	1.59	0	10

Note: Data for Germany: LINOS-2, N = 2,659 (1,386 women, 1,273 men); Data for the Netherlands: FSDP N = 2,678 (1,456 women, 1,222 men).

### Procedures

In discrete choice experiments, respondents choose (at least) one option out of multiple alternatives presented simultaneously to them within a so-called choice set. In these choice sets, different attributes vary experimentally in their levels. Therefore, discrete choice experiments are multi-factorial survey-experiments. The random allocation of the choice sets to respondents ensures independence of the respondent’s characteristics. Moreover, the composition of fictitious alternatives allows researchers to investigate people’s preferences for job offers independent of their current situation. This might potentially compromise the external validity; the extent to which results from discrete choice experiments can be generalized to actual job choice situations. Yet studies specifically investigating external validity of preferences or attitudes expressed in multi-factorial survey experiments conclude they are very similar to the preferences and attitudes respondents show in their real lives [72].

To create the three alternative job offers of our choice sets, we used D-efficient sampling strategies. This ensures efficient estimates of potential effects, as attributes are uncorrelated and balanced in their levels. For the sampling of the German choice sets, we used the user-written Stata ado *dcreate* [73]. We sampled 36 job offers from which we created 12 sets with alternative job offers. Respondents in the German survey were randomly faced with one of the 12 choice sets. Accordingly, we had a between-subjects design. Moreover, the order of the choices within each choice set was random; a specific job description was presented as the first alternative to one respondent and as the second or third alternative to another. Thus, we avoided primacy and recency effects [74].

To generate the experimental set-up for the Dutch survey, we used the sampling procedures implemented in SAS [75]. Again, we used the D-efficiency criterion to sample the alternatives and to combine them into choice sets. In this case, however, we developed a design in which every respondent answered three instead of one choice set. Therefore, we first sampled 90 alternative job offers. From these, we created ten sets of three choice sets. As before, each choice set consisted of three alternative job offers. Choice sets were once again randomly allocated, but this time, the order of appearance of the three sets every respondent rated was also random. This ensured that the levels of choice attributes and respondent characteristics were uncorrelated, and there were no order effects within the sets.

In both surveys, respondents could skip the task. In the German case, only 76 of the 2,741 respondents did not make a decision on a job offer. Thus, 97 percent of all choice sets were answered. Among the Dutch respondents, only 86 percent of all displayed choice sets were answered. This might indicate that the differences between job offers were more subtle in the Dutch case—due to the higher number of overall experimentally varied job offers (90 vs. 36 in the German data)—thus increasing the difficulty of the decision-making process.

### Measures

In the German version of the survey, our hypothetical job offers comprised five experimentally varied attributes: earnings, job security, opportunities for further training, scheduling flexibility, and prestige of the company. The Dutch version included a sixth attribute: the gender composition of the work team. All attributes had three levels (see Table 2). We varied earnings as average earnings, slightly above average earnings, and far above average earnings. We opted against job offers with under-average earnings as we assumed preferences for avoiding underpayment would dominate all other attributes. We also decided against concrete amounts of earnings to prevent respondents from comparing their actual earnings with those in the job offer. We measured job security by the employment duration specified in the job contract. Again, we varied three levels: a permanent contract, a 5-year contract, and a 2-year contract. In the third attribute, training opportunities, we distinguished between general training (e.g., distance learning, language courses) and work-specific training, both paid by the employer. General training measured investments in general human capital; work-specific training measured investments in specific human capital. The third level was no opportunities for further training. To capture preferences for family or care arrangements, we distinguished three types of scheduling flexibility that are commonly advertised in German job openings: flexible working hours with short-notice time off if needed, flexible working hours without extra time off if needed, and no flexibility in working hours at all. The reputation of the company offering the job was described as very good, average, or rather bad. The gender composition of the work team (only included in the Dutch data) varied between more men, more women, and about an equal share of men and women. S1 Table shows a choice set with all six attributes.

**Table 2:** Attributes and levels of the discrete choice experiment.

	Job attribute	Levels
1	Earnings	1: Far above average 2. Slightly above average 3. Average
2	Contract duration	1: Permanent contract 2: 5-year contract 3: 2-year contract
3	Training opportunities	1: General training paid by employer (e.g., distance learning, language courses) 2: Subject-specific training paid by employer 3: None
4	Family/care arrangements	1: Flexible work schedule with time off if needed 2: Flexible work schedule 3: None
5	Reputation of the company	1: Very good 2: Average 3: Rather bad
6	Gender composition (only in FSDP data)	1: More men 2: More women 3: About equal shares of men and women

### Data analytical approach

Our modelling was theoretically grounded in random utility theory [72, 76, 77]. The main idea of random utility theory is that people choose the option with maximum utility. Moreover, the overall utility can be decomposed into the partial utilities of each attribute that people value more or value less. Utility depends on aspects specific to the respondent (such as the current job situation or whether there are children in the household), on the job attributes presented in each job offer, and on an idiosyncratic error term. For example, in a model of a job offer choice in the German survey that depends on earnings (E), contract duration (C), training opportunities (T), family/care arrangements (F), reputation of the company (R), and the respondent’s gender (G), the utilities for the three choices would be the following:

$$U_{i1}=\alpha +\beta_{1}E_{i1}+\beta_{2}C_{i1}+\beta_{3}T_{i1}+\beta_{4}F_{i1}+\beta_{5}R_{i1}+\gamma G_{i}+\varepsilon_{i1};$$ (1)

$$U_{i2}=\alpha +\beta_{1}E_{i2}+\beta_{2}C_{i2}+\beta_{3}T_{i2}+\beta_{4}F_{i2}+\beta_{5}R_{i2}+\gamma G_{i}+\varepsilon_{i2};$$ (2)

$$U_{i3}=\alpha +\beta_{1}E_{i3}+\beta_{2}C_{i3}+\beta_{3}T_{i3}+\beta_{4}F_{i3}+\beta_{5}R_{i3}+\gamma G_{i}+\varepsilon_{i1}.$$ (3)

The choice for alternative 1 reveals that

$$U_{i1}-U_{i2}=\beta_{1}(E_{i1}-E_{i2})+\cdots +\beta_{5}(R_{i1}-R_{i2})+(\varepsilon_{i1}-\varepsilon_{i2})>0$$ (4)

and

$$U_{i1}-U_{i3}=\beta_{1}(E_{i1}-E_{i3})+\cdots +\beta_{5}(R_{i1}-R_{i3})+(\varepsilon_{i1}-\varepsilon_{i3})>0.$$ (5)

Obviously, the constant term (α) and the term for the respondent’s gender (γ*G*_i_) have fallen out of the comparison as they do not differ between the alternatives [78].

As people’s decisions on a job offer also depend on the displayed alternative offers, we estimated conditional logit models for the German and Dutch data. The conditional logit model (sometimes called multinomial logit model [78]) is appropriate for unlabeled and randomly ordered choices, as in our case (in many discrete choice applications, choices are labeled—e.g., choice of a specific mode of transportation or preference for a specific brand—and thus require strategies that allow estimation of alternative specific intercepts) [79, 80]. To test if respondent characteristics—such as gender—explained decisions for or against certain job offers, we included interaction terms. By doing this, we could test the theoretical assumption of gender differences for certain job attributes.

The structure of the Dutch survey was somewhat more complicated than the German one, as every respondent evaluated three choice sets. Therefore, we had 8,034 decisions nested in the 2,678 survey respondents. Re-analyses with panel-data mixed conditional logit models accounting for the data structure yielded results similar to those of the first choice set only. Therefore, we decided to report results the same way for the German and Dutch data.

In the following, we report all results as average semi-elasticities [81] and provide the corresponding tables with all estimates, along with information on model fit statistics in the Supporting information. For our data analysis, we used Stata 16.1, for the estimation of the average semi-elasticities, we used the user written *ado aextlogit* [82], for the presentation of results, we used the user-written *ados coefplot* [83], *estout* [84], and the scheme *plotplain* [85].

## Results

### Preferences for job characteristics in Germany and the Netherlands

**Fig 1:**
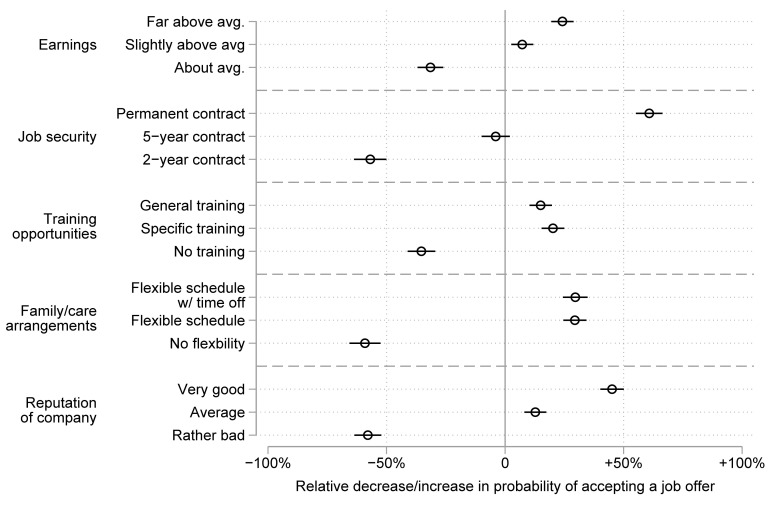
Preferences for job attributes among respondents in the German survey. The figure shows the relative decrease/increase in probabilities (with 95% confidence intervals) of choosing a job due to the respective job attribute.

Fig 1 shows the results of the discrete choice experiment for the German sample. The figure displays the average semi-elasticities for all levels in contrast to the grand mean. The grand mean reflects the decision probability for one of the three job offers if the respondent has no preferences for one job offer over the others. This base probability of about 1/3 is set to zero in the figures. Accordingly, a positive effect—displayed to the right of the zero line on the x-axis—indicates that people are, on average, more likely to choose a job offer if it includes the respective job attribute. An effect displayed to the left of the zero line indicates people are less likely to choose a job offer if it includes the respective job attribute. The whiskers represent the 95% confidence intervals. A confidence interval overlapping zero means the effect of the respective job attribute is statistically not significant.

Looking at the dimension earnings first, we see that the percentage increase in the (base) probability of choosing a job offer was 24.2 percent if earnings were far above average and if the job offered slightly above average earnings, the probability increased by 7.3 percent. However, if the job offer included only about average earnings, the (base) probability of choosing this offer decreased by 31.5 percent. Wald tests on the equality of these effects indicate that people significantly preferred earnings far above average over earnings slightly above average (χ^2^ = 19.11; p < .001), as well as far above average earnings over average earnings (χ^2^ = 146.78; p < .001), and slightly above average earnings over average earnings (χ^2^ = 72.40; p < .001). These results are in line with our theoretical expectations on the increasing utility of higher wages. Model 1 of S2 Table shows the corresponding semi-elasticities in the traditional way with a reference category—meaning that coefficients in all tables in the Supporting information must be interpreted with respect to the reference category instead of the base probability. For example, the coefficient of .557 for far above average earnings means that people are on average 55.7 percent more likely to choose a job offer displaying far above average earnings compared to a job offer with about average earnings (the reference category).

Second, people were, on average, 60.8 percent more likely to choose a job with a permanent contract and 56.9 percent less likely to choose a job lasting only 2-years. The effect of a 5-year contract was also slightly negative but not significant. Again, all differences between these levels of job security were statistically highly significant (permanent contract vs. 5-year contract: χ^2^ = 184.15; p < .001; 5-year contract vs. 2-year contract: χ^2^ = 80.54; p < .001).

Third, people preferred opportunities for further training over no training opportunities. Yet they did not differentiate between opportunities for general or specific training (χ^2^ = 1.80; p = .179) as theory would suggest, given the generalizability and convertibility of the former.

Fourth, scheduling flexibility was important. People were, on average, about 30 percent more likely to choose a job offering scheduling flexibility. Yet it seemed to make little difference if in addition to a flexible schedule, time off if needed was explicitly granted (χ^2^ = 0.01; p = .969). In contrast, people were 59.1 percent less likely to choose a job offer with no flexibility. This is in line with research suggesting employee preferences for more individualistic and flexible schedules.

Lastly, the reputation of the company made a difference in the decision process. People, on average, were much more likely to choose a job with a company with a very good reputation over one with a company with only an average reputation (χ^2^ = 68.18; p < .001) or a rather bad reputation (χ^2^ = 440.83; p < .001). Surprisingly, a very good company reputation was even more important than high earnings (χ^2^ = 49.53; p < .001), specific training opportunities (χ^2^ = 50.67; p < .001), or scheduling flexibility (χ^2^ = 20.62; p < .001).

**Fig 2:**
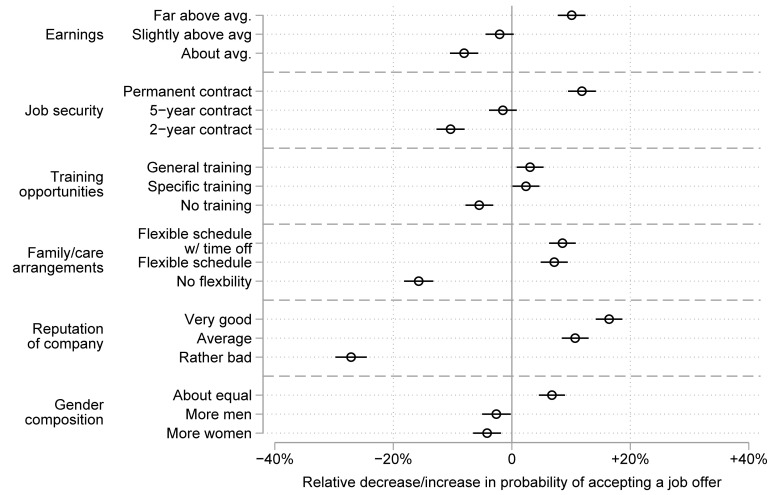
Preferences for job attributes among respondents in the Dutch survey. The figure shows the relative decrease/increase in probabilities (with 95% confidence intervals) of choosing a job due to the respective job attribute.

Fig 2 shows the results for the Dutch sample. The graphical presentation is similar to that for the German sample. Model 1 of S3 Table shows the respective coefficients and standard errors of the semi-elasticities when using specific reference categories.

Comparing Figs 1 and 2, we observe that the overall effect sizes in the Dutch data were smaller than in the German data, but the directions and the relative sizes of the effects were quite similar. Additional analyses restricting the survey to employed respondents, to identical choice sets across surveys, and to only the first of each respondent’s three decisions led to very similar results. This indicates that differences in study design did not account for the smaller effect sizes we found in the Dutch data. The preference structure was again very straightforward in terms of earnings, contract duration, further training, family/care arrangements, and reputation of the company. Yet in the Dutch case, the most important aspect driving the choice of a job offer was not job security but the reputation of the company. Nonetheless, job security was more important than high pay (χ^2^ = 172.97; p < .001) or scheduling flexibility (χ^2^ = 125.61; p < .001). Finally, with respect to the additional attribute included in the Dutch choice set—the gender composition of the work environment—people preferred working in workplaces with equal shares of men and women to working in male-dominated (χ^2^ = 21.38; p < .001) or female-dominated (χ^2^ = 30.88; p < .001) workplaces. People also showed a tendency to prefer male-dominated workplaces to female-dominated workplaces, but this difference was not statistically significant (χ^2^ = 0.53; p = .466).

### Gender differences in job preferences

**Fig 3:**
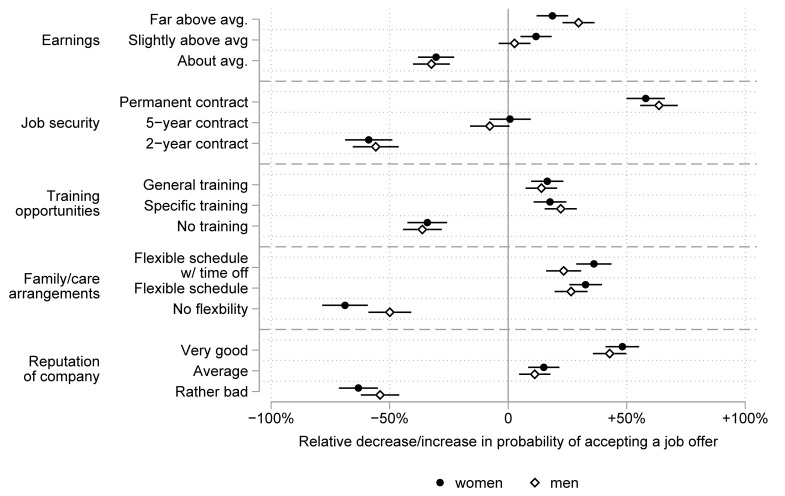
Preferences for job attributes among male and female respondents in the German survey. The figure shows the relative decrease/increase in probabilities (with 95% confidence intervals) of choosing a job due to the respective job attribute.

Fig 3 shows the results separately for men and women in the German sample. Again, we estimated the displayed average semi-elasticities in contrast to the grand mean from the coefficients of S2 Table (Model 2 and Model 3). We estimated separate models for men and women and a full interaction model to formally test for gender differences (Model 1 of S4 Table). The inspection of Fig 3 suggests men and women based their decisions for or against a job offer on the same job attributes, but they had slightly different priorities. For men, the option to receive wages far above average was a stronger predictor of choosing a job than for women. While men were more likely to choose jobs with high earnings than those with slightly above average earnings (χ^2^ = 23.42; p < .001), this differentiation did not matter as much to women (χ^2^ = 1.58; p = .208). Men and women did not seem to differ considerably in their preferences for jobs with average earnings. While this resonates with our theoretical expectation that men value extrinsic aspects of a job more than women, we observed no statistically significant gender differences in preferences for earnings far above average (χ^2^ = 1.92; p = .166) or slightly above average (χ^2^ = 0.63; p = .428). Again, the most important aspect of a job offer for both men and women was job security, and we observed no gender differences in preferences for job security. Turning to the further training opportunities, we observed a slight leaning of women towards general human capital and of men towards specific human capital. However, these gender differences were not statistically significant. The possibility of having a flexible work schedule was significantly more important for women than for men. Women were more likely to choose job offers with flexible working hours (χ^2^ = 5.88; p = .015) and flexible job offers with time off if needed (χ^2^ = 8.48; p = .004). This is largely in line with the literature highlighting different responsibilities of men and women in the arrangements of work and family life. Lastly, the reputation of the company seemed to be somewhat more important for women. As the reputation of the company can be considered an intrinsic job characteristic, this result resonates with our theoretical expectations of the gendered valuation of extrinsic and intrinsic job attributes. Yet formal tests showed no significant gender differences (χ^2^ = 2.33; p = .127).

When we compared the importance of the different job attributes separately for men and for women, we had some surprising results. Job security was the most important job attribute for men and women alike, but the preference order of the other job attributes was less clear. For men, a very good company reputation was even more important than high pay (χ^2^ = 10.04; p = .002). Surprisingly, for men, having a flexible schedule (χ^2^ = 0.46; p = .498) and specific training opportunities (χ^2^ = 2.36; p = .124) were as important as high pay. For women, jobs with a flexible schedule with (χ^2^ = 14.00; p < .001) or without (χ^2^ = 9.42; p = .002) time off if needed were more attractive than high-paying jobs. These results are in line with the theoretical expectation that women do most of the reconciling of work and family but are less supportive of the expectation that men will show preferences for extrinsic job attributes.

**Fig 4:**
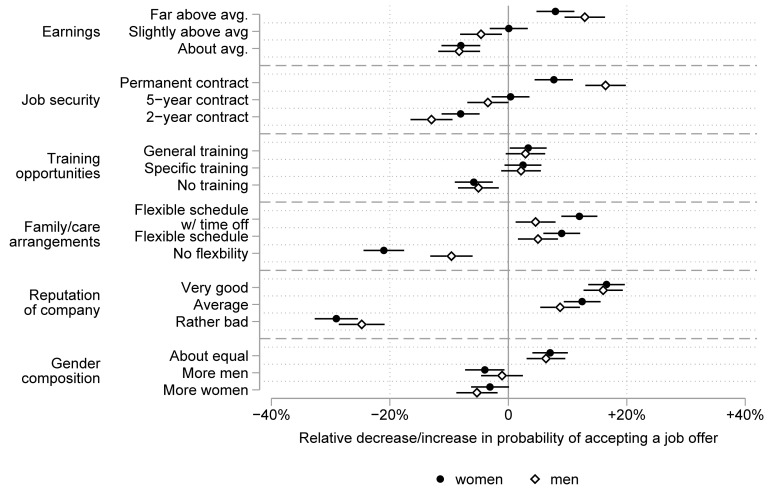
Preferences for job attributes among male and female respondents in the Dutch survey. The figure shows the relative decrease/increase in probabilities (with 95% confidence intervals) of choosing a job due to the respective job attribute.

Fig 4 shows the respective effects separately for male and female respondents. Model 2 and Model 3 of S3 Table display the respective estimated semi-elasticities and standard errors. Model 2 of S4 Table shows the full-interaction models that test for gender differences. The overall patterns shown in the German data in Fig 3 and the Dutch data in Fig 4 reveal remarkably similar preferences. As we found for the German respondents, among the Dutch respondents, high earnings and a permanent contract seemed to matter more for men. The formal test showed robust significant gender differences for a permanent contract (χ^2^ = 13.61; p < .001) and marginal significant differences for high earnings (χ^2^ = 3.37; p = .066). Scheduling flexibility (χ^2^ = 15.14; p < .001; with time off if needed: χ^2^ = 25.61; p < .001) and the reputation of the company (χ^2^ = 3.54; p = .060) were more important to women. With respect to family/care arrangements, it seemed women differentiated between the two types of scheduling flexibility. The probability of choosing a job increased by 12 percent if it included a flexible schedule with additional time of if needed, whereas it increased by 9 percent when “only” a flexible schedule was offered. This difference was not statistically significant, however (χ^2^ = 1.30; p = .253). Preferences for further training opportunities again resembled those we found in the German data. Yet the opportunity for further training did not affect women’s job choices significantly, and it affected men’s choices only slightly for general training. Results for preferences for the gender composition in the work environment were largely parallel for men and women: both seemed to prefer working with about equal shares of men and women. This result contradicts our theoretical expectation of homophily, namely, that men would prefer to work in male dominated and women in female dominated workplaces. There was a slight tendency among men to favor job offers from companies with more men over job offers from companies with more women and vice versa among women, but neither the difference among men (χ^2^ = 1.80; p = .179) nor the difference among women (χ^2^ = 0.09; p = .763) was statistically significant.

There were some remarkable cross-country gender differences. For Dutch men, high earnings were as important as a permanent contract (χ^2^ = 2.02; p = .155), a very good company reputation (χ^2^ = 0.03; p = .860), and a balanced gender composition of the work team (χ^2^ = 0.16; p = .686). In contrast to German men, for Dutch men, high pay was more important than training opportunities (χ^2^ = 16.27; p < .001) and scheduling flexibility (χ^2^ = 10.64; p < .001). For Dutch women, a very good company reputation was more important than high earnings (χ^2^ = 17.65; p < .001) and a permanent contract (χ^2^ = 16.69; p < .001). All other aspects, except for further training opportunities, were equally important for Dutch women. These results are again largely in line with the theoretical expectation that women do most of the reconciling of work and family and also speak to the greater importance men place on extrinsic job attributes.

Our theory section suggests that in Germany and the Netherlands, women are more likely than men to face the challenge of aligning work schedules with household needs. Much of the debate on the high shares of part-time working women has revolved around women’s need for scheduling flexibility if they care for children [46]. Therefore, we checked if children in the household moderated women’s preferences for jobs with scheduling flexibility. The respective full-interaction models are displayed in S5 and S6 Tables. For the German respondents (see S5 Table), the presence of children increased preferences for scheduling flexibility. However, this effect was entirely driven by women’s preferences for flexibility. German men showed no significantly increased preferences for flexible schedules if children were in the household (Model 3 of S5 Table). For the Dutch respondents (S6 Table), preferences for scheduling flexibility were also higher if children lived in the household. This effect seemed to be mostly driven by male respondents and likely reflected the less generous parental leave policies for fathers in the Netherlands.

### Robustness checks

To corroborate our results, we complemented our analyses with sensitivity analyses and robustness checks. As Dutch respondents completed three different choice sets, we re-analyzed the data with panel-data mixed conditional logit models to allow for correlations not only within but also across choice situations. In addition, we investigated whether the results changed when we restricted the data to the first choice per respondent. These sensitivity analyses yielded results similar to those presented above.

We checked whether the general patterns of our results held in two subgroup analyses. First, we investigated whether currently employed respondents differed in their job preferences from respondents who were not currently employed. The idea was that certain job characteristics, such as job security or flexibility, might be more important to employed respondents, as they might be more salient in their daily lives. S7 Table shows the full interaction model. Among the German respondents (Model 1), none of the interactions was significant, suggesting current employment status did not influence job preferences. However, two interactions were significant for the Dutch respondents (Model 2). For employed Dutch respondents, a permanent contract was somewhat more important, and a flexible schedule was somewhat less important than for the unemployed. Nevertheless, the general pattern of job preferences remained largely the same.

Second, we investigated if, among the employed, current job satisfaction affected the results. The idea was that those who were dissatisfied with their current job were more likely to be in the process of a job search and therefore would be faced with similar choices in their real lives. S8 Table shows a full interaction model with current job dissatisfaction as a moderator. We defined all employees who rated their job satisfaction on an 11-point scale below the country mean as dissatisfied. The interactions indicated that our reported results were solid. We only detected one significant interaction, whereby dissatisfied respondents had somewhat higher preferences for training opportunities.

## Discussion

In this study, we investigated which job attributes people consider more important when choosing a job. For this, we designed a discrete choice experiment in which we experimentally varied the levels of six job attributes. We expected to find positive effects of high earnings, permanent contracts, further training opportunities, scheduling flexibility, company reputation, and same gender working groups on the probability of choosing a job offer. In 2017, we implemented our discrete choice experiment in large-scale surveys in Germany and the Netherlands.

Even though the experimental design and the survey samples of the two studies differed considerably, our analyses revealed largely similar job preferences among German and Dutch respondents. More specifically, people’s decisions on a job offer were mostly driven by high job security in terms of a permanent contract, by high earnings, and by a very good company reputation. Yet we detected some notable cross-country differences in which job attribute people considered most important. While German respondents thought job security was the most important attribute, Dutch respondents pointed to the company reputation. Arguably, a permanent contract means more in terms of employment protection in Germany than the Netherlands. As German employment regulations set higher barriers for employers to fire employees without due cause, initial employment in Germany is increasingly on a fixed term basis. Because of the less strict employment regulations in the Netherlands, Dutch respondents might have associated permanent contracts less strongly with job security than German respondents. Somewhat surprisingly, in both countries, people thought high pay was less important than we had expected. Admittedly, we did not vary absolute pay levels; we only specified whether a specific job paid much more, slightly more, or about the same as similar jobs. In Germany, a permanent contract was more important than high pay; in the Netherlands, a good company reputation and a permanent contract were more important than high pay. This is in line with sociological theory’s suggestion that people’s job preferences are increasingly driven by other job aspects than high pay [11–15].

In more detailed analyses, we looked for gendered job preferences. As expected, while all respondents valued scheduling flexibility, it was more important for women than men. This supports our theoretical reasoning on traditional gender roles and is in line with recent research finding a considerable gender gap in household responsibilities [43–45]. To corroborate our interpretation of the finding, we investigated the moderating role of children in the household on job preferences. In both countries, we found more preference for scheduling flexibility if there were children in the household. Notably, in Germany, children in the household increased women’s preferences for scheduling flexibility but did not play a significant role in men’s preferences. This accords with recent research showing gender differences in scheduling flexibility and care responsibilities [86]. Interestingly, in the Netherlands, children in the household increased men’s but not women’s preferences for scheduling flexibility. In the Netherlands, women with children are more likely to work part time than men (and also more likely to work part time than German women), and for part-timers, scheduling flexibility may be less important. But this is only speculative. In-depth analysis and a thorough theoretical discussion are required to gain a better understanding of these country specific differences.

While our analyses confirmed our expectation that women would have stronger preferences for scheduling flexibility than men, we had mixed results for our broader expectations of men’s stronger preferences for extrinsic job attributes and women’s stronger preferences for intrinsic job attributes. Dutch men considered high pay more important than further training opportunities or scheduling flexibility, but German men showed no statistically significant preferences for either of these job attributes.

Lastly, the gender composition of the team, measured only in the Dutch sample, revealed that men and women both preferred to work in teams with a balanced share of men and women. This did not support our expectation of a preference for homophily among both men and women and might indicate that people dislike being in a minority position. Future studies should look into this in greater detail.

### Limitations

Our study contributes to the knowledge on preferences of employees for specific job aspects. Nonetheless, we must acknowledge some limitations. The first limitation is the lack of generalizability of our results. As discrete choice experiments are survey experiments with fictitious choices, it remains unclear whether people would use the same criteria and in the same way when making real-life decisions. Instead of actual behavior, we measured behavioral intentions. A recent study with a discrete choice design looking at job preferences of undergraduates found these preferences were largely related to actual job choices reported four years after graduation [11].

A second limitation was the difference in effect sizes for the two samples. The effects of the job attributes on the decision on a job offer were much stronger for the German than the Dutch sample. We believe the direction and relative importance of the specific dimensions are solid, and we offer a couple of possible explanations of the differences in effect sizes. First, these differences could reflect the different methodological approaches. We used more dimensions, more job offers, and more decisions per respondent in the Dutch survey. Consequently, the decisions might have been harder for the Dutch respondents, thereby leading to smaller effect sizes. The higher number of missing values on job choices among the Dutch respondents suggests this possibility. Second, the differences might indicate that the phrasing of the different levels of attributes captured the theoretical ideas better in the German context. Third, there may be actual country differences in people’s preference structures related to national norms and/or labor market structures. We encourage scholars to investigate job preference structures from a cross-country comparative perspective to probe this issue.

## Conclusions

In this paper, we have shown how the use of discrete choice sets might shed light on people’s preferences for certain job attributes. While our results are largely in line with findings from previous observational studies, they suggest job-seeking behavior is not solely driven by a desire to maximize earnings. Moreover, our finding of gendered preferences suggests women value different job characteristics than men.

We have simply given an overview, and many more questions could be answered with our design. A promising approach would be to focus more specifically on a single job attribute or the social context of respondents. For example, our robustness checks revealed that respondents who were dissatisfied in their current job had a higher preference for training opportunities. Intuitively this makes sense; dissatisfied people may seek avenues to leave their present job. Corroboration of this interpretation requires a sound theoretical foundation and the formulation of specific hypotheses that take different potential causes of job dissatisfaction into account. Our complementary analyses revealed that children in the household moderated respondents’ preferences for scheduling flexibility but affected men and women differently in Germany and the Netherlands. Future studies with a strong theoretical basis focusing on country level explanations and using in-depth analyses could develop our understanding of these country specific peculiarities.

## Supporting information

S1 Table Example choice set with six job attributes. (PDF)

S2 Table Main effects for German respondents choosing a job offer. (PDF)

S3 Table Main effects for Dutch respondents choosing a job offer. (PDF)

S4 Table Full-interaction models w/ respondent’s gender as moderator. (PDF)

S5 Table Full-interaction models for German respondents w/ children in household as moderator. (PDF)

S6 Table Full-interaction models for Dutch respondents w/ children in household as moderator. (PDF)

S7 Table Full-interaction models w/ respondent’s current employment status as moderator. (PDF)

S8 Table Full-interaction models w/ respondent’s current job satisfaction as moderator. (PDF)
