# Supplementary material for: Preferences for work arrangements: A discrete choice experiment
Source: PLoS One. 2021 Jul 12;16(7):e0254483. doi: 10.1371/journal.pone.0254483 (PMC8274907; doi:10.1371/journal.pone.0254483)
Supplement: S1 Table — (PDF) [file pone.0254483.s001.pdf]

S1 Table. Example choice set with six job attributes.

**Here we show you three job offers. Imagine—independent of your current employment situation—that you must decide for one of these three job offers. Which one would you choose?**<br><br>Note: The descriptions of these offers are very general and differ only in a few aspects. Please assume that these offers do not differ in other aspects than those depicted here.			
	**Job offer 1**	**Job offer 2**	**Job offer 3**
**Training opportunities**	None	General training paid by employer (e.g., distance learning, language courses)	Work-specific training paid by employer
**Salary/wages**	Average	Somewhat above average	Far above average
**Contract duration**	Permanent	5 years	2 years
**Family/care arrangements**	Flexible working hours	None	Flexible working hours and time off if needed
**Reputation of the company**	Very good	Rather bad	Average
**Gender composition of the work team**	More women	More men	Equal share of men and women
**Your choice**	☐	☐	☐
