# Supplementary material for: Preferences for work arrangements: A discrete choice experiment
Source: PLoS One. 2021 Jul 12;16(7):e0254483. doi: 10.1371/journal.pone.0254483 (PMC8274907; doi:10.1371/journal.pone.0254483)
Supplement: S2 Table — (PDF) [file pone.0254483.s002.pdf]

**S2 Table. Main effects for German respondents choosing a job offer.**

| | (1) All (GER) | | (2) Women (GER) | | (3) Men (GER) | |
|---|---|---|---|---|---|---|
| | Semi-elasticity | Standard error | Semi-elasticity | Standard error | Semi-elasticity | Standard error |
| Earnings: | | | | | | |
| About average (ref.) | ref. | | ref. | | ref. | |
| Far above average | .557*** | (.046) | .490*** | (.065) | .621*** | (.066) |
| Slightly above average | .387*** | (.046) | .422*** | (.064) | .351*** | (.066) |
| Job security: | | | | | | |
| 2-year contract (ref.) | ref. | | ref. | | ref. | |
| Permanent contract | 1.177*** | (.056) | 1.168*** | (.081) | 1.195*** | (.080) |
| 5-year contract | .529*** | (.059) | .596*** | (.086) | .481*** | (.083) |
| Training opportunities: | | | | | | |
| No training (ref.) | ref. | | ref. | | ref. | |
| General training | .503*** | (.048) | .506*** | (.069) | .502*** | (.068) |
| Specific training | .555*** | (.049) | .517*** | (.070) | .584*** | (.069) |
| Family/care arrangements: | | | | | | |
| No flexibility (ref.) | ref. | | ref. | | ref. | |
| Flexible schedule w/ time off | .887*** | (.055) | 1.049*** | (.080) | .733*** | (.076) |
| Flexible schedule | .885*** | (.053) | 1.015*** | (.076) | .764*** | (.073) |
| Reputation of the company: | | | | | | |
| Rather bad (ref.) | ref. | | ref. | | ref. | |
| Very good | 1.030*** | (.049) | 1.113*** | (.071) | .968*** | (.070) |
| Average | .706*** | (.046) | .782*** | (.067) | .652*** | (.066) |
| Log-likelihood (full model) | -2022.82 | | -1013.12 | | -987.65 | |
| Likelihood ratio $\chi^2$ | 1809.96 | | 1019.12 | | 821.76 | |
| Prob > LR | <.001 | | <.001 | | <.001 | |
| Respondents | 2659 | | 1386 | | 1273 | |
| Job offers | 7977 | | 4158 | | 3819 | |

*Note: LINOS-2 data. Conditional logit models. Displayed are average semi-elasticities and standard errors in parentheses.*

$^{*}$ $p < .05$, $^{**}$ $p < .01$, $^{***}$ $p < .001$
