# Supplementary material for: Preferences for work arrangements: A discrete choice experiment
Source: PLoS One. 2021 Jul 12;16(7):e0254483. doi: 10.1371/journal.pone.0254483 (PMC8274907; doi:10.1371/journal.pone.0254483)
Supplement: S3 Table — (PDF) [file pone.0254483.s003.pdf]

S3 Table. Main effects for Dutch respondents choosing a job offer.

	(1) All (NL)		(2) Women (NL)		(3) Men (NL)	
	Semi-elasticity	Standard error	Semi-elasticity	Standard error	Semi-elasticity	Standard error
Earnings:						
About average (ref.)	ref.		ref.		ref.	
Far above average	.182***	(.021)	.160***	(.029)	.212***	(.030)
Slightly above average	.060**	(.021)	.081**	(.029)	.037	(.031)
Job security:						
2-year contract (ref.)	ref.		ref.		ref.	
Permanent contract	.222***	(.021)	.157***	(.029)	.294***	(.031)
5-year contract	.088***	(.021)	.084**	(.028)	.095**	(.031)
Training opportunities:						
No training (ref.)	ref.		ref.		ref.	
General training	.086***	(.020)	.092**	(.028)	.079**	(.030)
Specific training	.079***	(.020)	.083**	(.028)	.072*	(.030)
Family/care arrangements:						
No flexibility (ref.)	ref.		ref.		ref.	
Flexible schedule w/ time off	.243***	(.021)	.330***	(.029)	.142***	(.031)
Flexible schedule	.229***	(.021)	.300***	(.029)	.146***	(.031)
Reputation of the company:						
Rather bad (ref.)	ref.		ref.		ref.	
Very good	.435***	(.022)	.456***	(.031)	.408***	(.032)
Average	.378***	(.022)	.415***	(.031)	.335***	(.033)
Gender composition of work team:						
More women (ref.)	ref.		ref.		ref.	
About equal	.109***	(.020)	.101***	(.027)	.117***	(.029)
More men	.016	(.022)	-.009	(.029)	.042	(.032)
Log-likelihood (full model)	-8439.85		-4561.04		-3852.57	
Likelihood ratio χ^2	772.79		475.39		349.88	
Prob > LR	<.001		<.001		<.001	
Respondents	2678		1456		1222	
Job offers	24102		13104		10998	

Note: FSDP data. Conditional logit models. Displayed are average semi-elasticities and standard errors in parentheses.

* $p < .05$, ** $p < .01$, *** $p < .001$
