# Supplementary material for: Preferences for work arrangements: A discrete choice experiment
Source: PLoS One. 2021 Jul 12;16(7):e0254483. doi: 10.1371/journal.pone.0254483 (PMC8274907; doi:10.1371/journal.pone.0254483)
Supplement: S4 Table — (PDF) [file pone.0254483.s004.pdf]

S4 Table. Full-interaction models w/ respondent's gender as moderator.

	(1) GER		(2) NL	
	Semi-elasticity	Standard error	Semi-elasticity	Standard error
Earnings:				
About average (ref.)	ref.		ref.	
Far above average	.619***	(.066)	.277***	(.035)
Slightly above average	.350***	(.065)	.056	(.035)
Job security:				
2-year contract (ref.)	ref.		ref.	
Permanent contract	1.192***	(.079)	.370***	(.035)
5-year contract	.480***	(.082)	.123***	(.035)
Training opportunities:				
No training (ref.)	ref.		ref.	
General training	.501***	(.068)	.103**	(.035)
Specific training	.582***	(.068)	.082*	(.034)
Family/care arrangements:				
Flexible schedule (ref.)	ref.		ref.	
Flexible schedule w/ time off	.732***	(.076)	.176***	(.035)
Flexible schedule	.763***	(.073)	.186***	(.035)
Reputation of the company:				
Rather bad (ref.)	ref.		ref.	
Very good	.966***	(.069)	.507***	(.036)
Average	.651***	(.066)	.411***	(.036)
Gender composition of the company:				
More women (ref.)			ref.	
About equal			.154***	(.033)
More men			.052	(.035)
Interactions w/ respondent's gender:				
Earnings:				
Far above average × Woman	-.128	(.092)	-.086	(.047)
Slightly above average × Woman	.073	(.092)	.040	(.047)
Job security:				
Permanent contract × Woman	-.021	(.112)	-.173***	(.047)
5-year contract × Woman	.118	(.119)	-.020	(.048)
Training opportunities:				
General training × Woman	.006	(.097)	.018	(.047)
Specific training × Woman	-.064	(.098)	.019	(.047)
Family/care arrangements:				
Flexible schedule w/ time off × Woman	.320**	(.110)	.242***	(.048)
Flexible schedule × Woman	.254*	(.105)	.185***	(.048)
Reputation of the company:				
Very good × Woman	.149	(.098)	.049	(.049)
Average × Woman	.133	(.093)	.092	(.049)
Gender composition of the company:				
About equal × Woman			-.019	(.045)
More men × Woman			-.063	(.048)
Log-likelihood (full model)	-2000.77		-11502.82	
Likelihood ratio χ^2	1840.88		1035.17	
Prob > LR	<.001		<.001	
Respondents	2659		2678	
Job offers	7977		24102	

Note: LINOS-2 data (model 1) and FSDP data (model 2). Conditional logit models. Displayed are average semi-elasticities and standard errors in parentheses.

* *p < .05*, ** *p < .01*, *** *p < .001*
