# Supplementary material for: Preferences for work arrangements: A discrete choice experiment
Source: PLoS One. 2021 Jul 12;16(7):e0254483. doi: 10.1371/journal.pone.0254483 (PMC8274907; doi:10.1371/journal.pone.0254483)
Supplement: S5 Table — (PDF) [file pone.0254483.s005.pdf]

S5 Table. Full-interaction models for German respondents w/ children in household as moderator.

	(1) All (GER)		(2) Women (GER)		(3) Men (GER)	
	Semi-elasticity	Standard error	Semi-elasticity	Standard error	Semi-elasticity	Standard error
Earnings:						
About average (ref.)	ref.		ref.		ref.	
Far above average	.584***	(.069)	.516***	(.099)	.651***	(.098)
Slightly above average	.445***	(.067)	.506***	(.097)	.394***	(.096)
Job security:						
2-year contract (ref.)	ref.		ref.		ref.	
Permanent contract	1.249***	(.085)	1.249***	(.123)	1.258***	(.120)
5-year contract	.647***	(.087)	.748***	(.126)	.577***	(.125)
Training opportunities:						
No training (ref.)	ref.		ref.		ref.	
General training	.529***	(.068)	.460***	(.096)	.598***	(.098)
Specific training	.532***	(.071)	.468***	(.099)	.591***	(.102)
Family/care arrangements:						
Flexible schedule (ref.)	ref.		ref.		ref.	
Flexible schedule w/ time off	.763***	(.077)	.896***	(.112)	.646***	(.109)
Flexible schedule	.749***	(.073)	.836***	(.103)	.679***	(.106)
Reputation of the company:						
Rather bad (ref.)	ref.		ref.		ref.	
Very good	1.028***	(.072)	1.159***	(.105)	.924***	(.101)
Average	.691***	(.069)	.816***	(.101)	.592***	(.097)
Interactions w/ children in household:						
Earnings:						
Far above average × Children in HH	-.130	(.104)	-.176	(.147)	-.102	(.150)
Slightly above average × Children in HH	-.133	(.103)	-.224	(.145)	-.090	(.149)
Job security:						
Permanent contract × Children in HH	-.116	(.127)	-.221	(.182)	-.017	(.183)
5-year contract × Children in HH	-.159	(.134)	-.312	(.196)	-.043	(.189)
Training opportunities:						
General training × Children in HH	-.112	(.110)	.078	(.162)	-.257	(.154)
Specific training × Children in HH	-.044	(.110)	.038	(.163)	-.101	(.154)
Family/care arrangements:						
Flexible schedule w/ time off × Children in HH	.275*	(.126)	.395*	(.187)	.176	(.176)
Flexible schedule × Children in HH	.299*	(.120)	.488**	(.180)	.132	(.166)
Reputation of the company:						
Very good × Children in HH	-.032	(.111)	-.091	(.162)	.022	(.157)
Average × Children in HH	-.030	(.105)	-.080	(.151)	.008	(.150)
Log-likelihood (full model)	-1604.78		-801.97		-777.75	
Likelihood ratio χ^2	1428.78		823.99		643.92	
Prob > LR	<.001		<.001		<.001	
Respondents	2106		1105		1001	
Job offers	6318		3315		3003	

Note: LINOS-2 data. Conditional logit models. Displayed are average semi-elasticities and standard errors in parentheses.
* $p < .05$, ** $p < .01$, *** $p < .001$
