# Supplementary material for: Preferences for work arrangements: A discrete choice experiment
Source: PLoS One. 2021 Jul 12;16(7):e0254483. doi: 10.1371/journal.pone.0254483 (PMC8274907; doi:10.1371/journal.pone.0254483)
Supplement: S6 Table — (PDF) [file pone.0254483.s006.pdf]

S6 Table. Full-interaction models for Dutch respondents w/ children in household as moderator.

	(1) All (NL)		(2) Women (NL)		(3) Men (NL)	
	Semi-elasticity	Standard error	Semi-elasticity	Standard error	Semi-elasticity	Standard error
Earnings:						
About average (ref.)	ref.		ref.		ref.	
Far above average	.159***	(.036)	.155**	(.050)	.177***	(.052)
Slightly above average	.054	(.036)	.081	(.050)	.026	(.054)
Job security:						
2-year contract (ref.)	ref.		ref.		ref.	
Permanent contract	.182***	(.036)	.110*	(.049)	.261***	(.053)
5-year contract	.069	(.036)	.061	(.048)	.079	(.053)
Training opportunities:						
No training (ref.)	ref.		ref.		ref.	
General training	.036	(.035)	-.006	(.049)	.080	(.051)
Specific training	.040	(.035)	.054	(.048)	.024	(.052)
Family/care arrangements:						
Flexible schedule (ref.)	ref.		ref.		ref.	
Flexible schedule w/ time off	.262***	(.036)	.386***	(.050)	.122*	(.052)
Flexible schedule	.206***	(.037)	.313***	(.051)	.086	(.053)
Reputation of the company:						
Rather bad (ref.)	ref.		ref.		ref.	
Very good	.425***	(.038)	.531***	(.053)	.302***	(.055)
Average	.380***	(.038)	.466***	(.054)	.279***	(.055)
Gender composition of the company:						
More women (ref.)	ref.		ref.		ref.	
About equal	.063	(.034)	.056	(.045)	.072	(.050)
More men	-.048	(.037)	-.090	(.051)	.002	(.054)
Interactions w/ children in household:						
Earnings:						
Far above average × Children in HH	-.023	(.053)	-.082	(.072)	.049	(.078)
Slightly above average × Children in HH	-.046	(.053)	-.056	(.072)	-.061	(.082)
Job security:						
Permanent contract × Children in HH	.081	(.053)	.114	(.072)	.057	(.079)
5-year contract × Children in HH	.064	(.053)	.061	(.070)	.084	(.081)
Training opportunities:						
General training × Children in HH	.038	(.052)	.093	(.070)	-.031	(.078)
Specific training × Children in HH	.049	(.052)	.017	(.070)	.085	(.078)
Family/care arrangements:						
Flexible schedule w/ time off × Children in HH	.055	(.054)	.039	(.073)	.042	(.081)
Flexible schedule × Children in HH	.133*	(.054)	.089	(.074)	.172*	(.080)
Reputation of the company:						
Very good × Children in HH	.004	(.056)	-.101	(.076)	.124	(.083)
Average × Children in HH	-.032	(.056)	-.087	(.077)	.040	(.084)
Gender composition of the company:						
About equal × Children in HH	.079	(.050)	.062	(.067)	.100	(.077)
More men × Children in HH	.154**	(.054)	.123	(.074)	.199*	(.081)
Log-likelihood (full model)	-5271.79		-2916.47		-2320.68	
Likelihood ratio χ^2	508.46		367.63		210.11	
Prob > LR	<.001		<.001		<.001	
Respondents	1676		940		736	
Job offers	15090		8466		6624	

Note: FSDP data. Conditional logit models. Displayed are average semi-elasticities and standard errors in parentheses.
* p < .05, ** p < .01, *** p < .001
