# Supplementary material for: Preferences for work arrangements: A discrete choice experiment
Source: PLoS One. 2021 Jul 12;16(7):e0254483. doi: 10.1371/journal.pone.0254483 (PMC8274907; doi:10.1371/journal.pone.0254483)
Supplement: S7 Table — (PDF) [file pone.0254483.s007.pdf]

S7 Table. Full-interaction models w/ respondent's current employment status as moderator.

	(1) GER		(2) NL	
	Semi-elasticity	Standard error	Semi-elasticity	Standard error
Earnings:				
About average (ref.)	ref.		ref.	
Far above average	.466***	(.133)	.154***	(.035)
Slightly above average	.327*	(.136)	.092**	(.035)
Job security:				
2-year contract (ref.)	ref.		ref.	
Permanent contract	1.029***	(.163)	.152***	(.034)
5-year contract	.415*	(.178)	.076*	(.034)
Training opportunities:				
No training (ref.)	ref.		ref.	
General training	.421**	(.156)	.052	(.034)
Specific training	.445**	(.153)	.075*	(.034)
Family/care arrangements:				
Flexible schedule (ref.)	ref.		ref.	
Flexible schedule w/ time off	1.175***	(.180)	.310***	(.035)
Flexible schedule	1.094***	(.169)	.279***	(.036)
Reputation of the company:				
Rather bad (ref.)	ref.		ref.	
Very good	1.098***	(.147)	.464***	(.037)
Average	.767***	(.141)	.420***	(.037)
Gender composition of the company:				
More women (ref.)			ref.	
About equal			.081*	(.032)
More men			-.052	(.036)
Interactions w/ employment status:				
Earnings:				
Far above average × Currently employed	.109	(.141)	.043	(.043)
Slightly above average × Currently employed	.070	(.144)	-.054	(.044)
Job security:				
Permanent contract × Currently employed	.174	(.173)	.109*	(.043)
5-year contract × Currently employed	.134	(.189)	.021	(.043)
Training opportunities:				
General training × Currently employed	.095	(.164)	.054	(.042)
Specific training × Currently employed	.128	(.161)	.009	(.042)
Family/care arrangements:				
Flexible schedule w/ time off × Currently employed	-.320	(.189)	-.110*	(.044)
Flexible schedule × Currently employed	-.237	(.178)	-.083	(.044)
Reputation of the company:				
Very good × Currently employed	-.072	(.156)	-.051	(.046)
Average × Currently employed	-.063	(.149)	-.071	(.046)
Gender composition of the company:				
About equal × Currently employed			.047	(.041)
More men × Currently employed			.110*	(.045)
Log-likelihood (full model)	-2016.53		-8421.87	
Likelihood ratio χ^2	1822.54		808.77	
Prob > LR	<.001		<.001	
Respondents	2659		2678	
Job offers	7977		24102	

Note: LINOS-2 data (model 1) and FSDP data (model 2). Conditional logit models. Displayed are average semi-elasticities and standard errors in parentheses.

* $p < .05$, ** $p < .01$, *** $p < .001$
