# Supplementary material for: Preferences for work arrangements: A discrete choice experiment
Source: PLoS One. 2021 Jul 12;16(7):e0254483. doi: 10.1371/journal.pone.0254483 (PMC8274907; doi:10.1371/journal.pone.0254483)
Supplement: S8 Table — (PDF) [file pone.0254483.s008.pdf]

S8 Table. Full-interaction models w/ respondent's current job satisfaction as moderator.

	(1) GER		(2) NL	
	Semi-elasticity	Standard error	Semi-elasticity	Standard error
Earnings:				
About average (ref.)	ref.		ref.	
Far above average	.566***	(.054)	.173***	(.029)
Slightly above average	.373***	(.053)	.016	(.029)
Job security:				
2-year contract (ref.)	ref.		ref.	
Permanent contract	1.195***	(.067)	.250***	(.029)
5-year contract	.571***	(.068)	.073*	(.029)
Training opportunities:				
No training (ref.)	ref.		ref.	
General training	.457***	(.056)	.061*	(.028)
Specific training	.545***	(.056)	.053	(.028)
Family/care arrangements:				
Flexible schedule (ref.)	ref.		ref.	
Flexible schedule w/ time off	.865***	(.063)	.190***	(.029)
Flexible schedule	.857***	(.060)	.190***	(.029)
Reputation of the company:				
Rather bad (ref.)	ref.		ref.	
Very good	1.027***	(.057)	.396***	(.030)
Average	.697***	(.054)	.330***	(.031)
Gender composition of the company:				
More women (ref.)			ref.	
About equal			.127***	(.028)
More men			.065*	(.030)
Interactions w/ job satisfaction:				
Earnings:				
Far above average × Currently dissatisfied	.002	(.115)	.095	(.064)
Slightly above average × Currently dissatisfied	.055	(.115)	.103	(.066)
Job security:				
Permanent contract × Currently dissatisfied	-.097	(.133)	.049	(.065)
5-year contract × Currently dissatisfied	-.268	(.156)	.109	(.064)
Training opportunities:				
General training × Currently dissatisfied	.331*	(.133)	.211***	(.064)
Specific training × Currently dissatisfied	.193	(.136)	.148*	(.065)
Family/care arrangements:				
Flexible schedule w/ time off × Currently dissatisfied	.095	(.147)	.043	(.065)
Flexible schedule × Currently dissatisfied	.131	(.143)	.030	(.065)
Reputation of the company:				
Very good × Currently dissatisfied	-.065	(.121)	.056	(.069)
Average × Currently dissatisfied	-.004	(.116)	.070	(.069)
Gender composition of the company:				
About equal × Currently dissatisfied			-.009	(.061)
More men × Currently dissatisfied			-.047	(.066)
Log-likelihood (full model)	-1877.36		-5350.50	
Likelihood ratio χ^2	1690.01		513.63	
Prob > LR	<.001		<.001	
Respondents	2478		1702	
Job offers	7434		15312	

Note: LINOS-2 data (model 1) and FSDP data (model 2). Conditional logit models. Displayed are average semi-elasticities and standard errors in parentheses.

* $p < .05$, ** $p < .01$, *** $p < .001$
